# Metatranscriptomics-based investigation of bacterial community dynamics across a dissolved organic matter gradient in southern Lake Michigan

**DOI:** 10.1128/aem.00263-26

**Published:** 2026-03-20

**Authors:** Adit Chaudhary, Hui Lin, Laodong Guo, Rachel Poretsky

**Affiliations:** 1 Department of Biological Sciences, University of Illinois at Chicago 166975, Chicago, Illinois, USA; 2 School of Freshwater Sciences, University of Wisconsin-Milwaukee 543317, Milwaukee, Wisconsin, USA; Michigan State University, East Lansing, Michigan, USA

**Keywords:** Lake Michigan, bacterioplankton, dissolved organic matter, metatranscriptomics, biogeochemical cycles, lakes, nutrient limitation

## Abstract

Bacterial communities in freshwater ecosystems play a critical role in biogeochemical and food web dynamics. However, our understanding of environmental controls on bacterial community function, particularly in large lakes, is limited. We characterized Lake Michigan water chemistry, bacterial community function, and substrate preferences to better understand bacteria-water chemistry relationships. Using metatranscriptomics, we investigated bacterioplankton gene expression in surface waters across a nearshore-to-offshore transect during 2017–2018. Additionally, we measured nutrients, dissolved organic carbon (DOC), chromophoric dissolved organic matter (DOM), as well as changes in major fluorescent DOM components across this transect. The results highlighted similarities in inorganic N and P measurements and a minor reduction in DOC levels from nearshore to offshore. However, significant changes in the composition of DOM were observed across the transect, including a higher presence of terrestrially derived and high-molecular-weight DOM in the nearshore. These differences in DOM quality were associated with the differential expression of several gene families between nearshore and offshore bacterioplankton. Notably, genes involved in the acquisition of various DOM, N, and P substrates, including peptidases, proteases, and transporter genes for amino acids, nucleobases, sugars, urea, and inorganic phosphate, were over-represented in the offshore bacterioplankton. A focused analysis of all the transporter gene expression for C, N, and P substrates revealed similar trends—higher expression of DOM transporter genes in the offshore versus nearshore. When viewed in the context of changing DOM quality across the transect, these results imply that offshore bacterial communities are more substrate-limited (particularly C) than in nearshore and are investing more energy in acquiring DOM substrates.

**IMPORTANCE:**

Various environmental, geological, and climatic factors influence bacterial community dynamics in freshwater ecosystems in complex and interactive ways. It thus becomes challenging in microbial ecology studies to disentangle the specific effects of these factors on microbial community function. Spatial environmental gradients in large lake ecosystems can provide a unique opportunity to test important questions about bacterial function and water chemistry relationships in a relatively consistent geological and climatic framework. Lake Michigan, one of the five largest lakes in the world, is one such example. The lake has witnessed significant ecological changes in the last few decades, and the impact of these changes on the physico-chemical environment and bacterioplankton function is not fully understood. In a relatively novel approach for freshwater systems, this study assesses Lake Michigan bacterial metabolism using robust transcriptomics techniques in the context of rich environmental data, including characterization of the lake chromophoric DOM and fluorescent DOM pool.

## INTRODUCTION

Despite covering a small fraction of Earth’s surface, freshwater ecosystems contribute significantly to regional and global carbon budgets (1, 2) and provide valuable ecosystem services to humans and the biodiversity inhabiting these systems. Heterotrophic bacterioplankton play a critical role in the biogeochemical cycling and food web dynamics in freshwater lake ecosystems by assimilating components of the dissolved organic matter (DOM) pool, a key process of the microbial loop that links bacterial metabolism to the lower food web in the lake. Several geological and environmental factors play an important role in influencing bacterial production and community dynamics in lake ecosystems. These factors include pH, temperature, lake geography and landscape position, primary production, nutrients, and DOM characteristics (3–7). However, these factors can have complex direct and interactive effects on lake bacterial community structure and function, and often, it can be challenging to disentangle the specific effects of different environmental variables on bacterial community dynamics. Within a relatively homogeneous geological framework, large lakes often exhibit environmental gradients and complex hydrodynamic circulation patterns similar to marine ecosystems (8). In particular, coastal-to-offshore spatial gradients can provide a promising study system to investigate bacteria-water chemistry relationships with limited confounding effects of other abiotic factors. Several studies have explored the effect of limiting nutrients or other environmental parameters on bacterial community function in marine coastal-to-offshore gradients (9–11), but similar investigations in large lake systems have been relatively limited (12–14).

Lake Michigan is one of the five largest freshwater lakes in the world and the second largest of the Laurentian Great Lakes by volume (15). Its ecology and trophic status have shifted from meso-oligotrophic to oligotrophic in the last few decades following reductions in total phosphorus loading and the expansion of invasive dreissenid mussels into its deeper offshore regions (16, 17). Previous studies in Lake Michigan have investigated spatiotemporal dynamics in bacterial abundance, production, respiration, diversity, and activity (14, 18–24). The results have highlighted broad similarities in bacterial abundance and community diversity across different regions of the lake, but also shown higher bacterial production and respiration rates in the nearshore versus offshore. Overall, the results signify the importance of autochthonous primary production as the dominant carbon source for bacterial production in the pelagic zone and provide evidence for the potential role of allochthonous riverine inputs in nearshore bacterial metabolism (18). Zhou et al. (25) reported that DOM in open Great Lakes originates mostly from autochthonous sources, with lower humification index (HIX) and median biological index (BIX) values in Lake Michigan, suggesting higher bioreactivity of DOM in open Lake Michigan. The decline in the spring diatom bloom due to the invasive dreissenid mussel proliferation has likely resulted in a major loss of annual labile DOM source for bacterial secondary production in Lake Michigan (16, 26). However, the nearshore regions receive large tributary inputs in the southern half of the lake that can offset some of the mussel filtering effects by bringing in terrestrial DOM and nutrient subsidies (27). Lake internal hydrodynamic processes, which include cyclonic circulation in the southern half of Lake Michigan and alongshore currents on the southeastern coast, can retard the mixing of nearshore and offshore waters (8, 28, 29). All these factors likely contribute to differences in water chemistries between coastal and offshore regions. Robust characterizations of bacterioplankton ecophysiology—both composition and activity—in southern Lake Michigan can provide valuable insights into microbial metabolism during times of significant ecological changes in this important freshwater resource.

In our previous study, we provided preliminary evidence of similarities and differences in the active (mRNA-based) bacterial community between nearshore and offshore southern Lake Michigan in a single season (22). In addition, we evaluated the relative importance of terrestrially derived DOM in supporting bacterial metabolism in the lake. Here, we extend the previous work with a more comprehensive survey of bacterioplankton dynamics in 2017 and 2018 across the same nearshore-to-offshore transect near the mouth of the Kalamazoo River, one of the largest tributaries to Lake Michigan. Using metatranscriptomics, we assessed bacterial community gene expression profiles in summer 2017–2018 and spring 2018 for nearshore (<5 km from shore) and offshore sites (~10–50 km from shore). In addition, we also measured dissolved nutrient concentrations (DOC, orthophosphate, and nitrate/nitrite) and characterized the bulk DOM pool using fluorescence excitation-emission matrix (EEM) across the transect. Our main goals of this study were to (i) characterize the differences and similarities in the water chemistry of nearshore and offshore Lake Michigan, (ii) compare microbial heterotrophic metabolism between the two regions in the context of this environmental data, and (iii) assess the substrate preferences for lake bacterioplankton across this environmental gradient.

## MATERIALS AND METHODS

### Sample collection and processing

**Fig 1:**
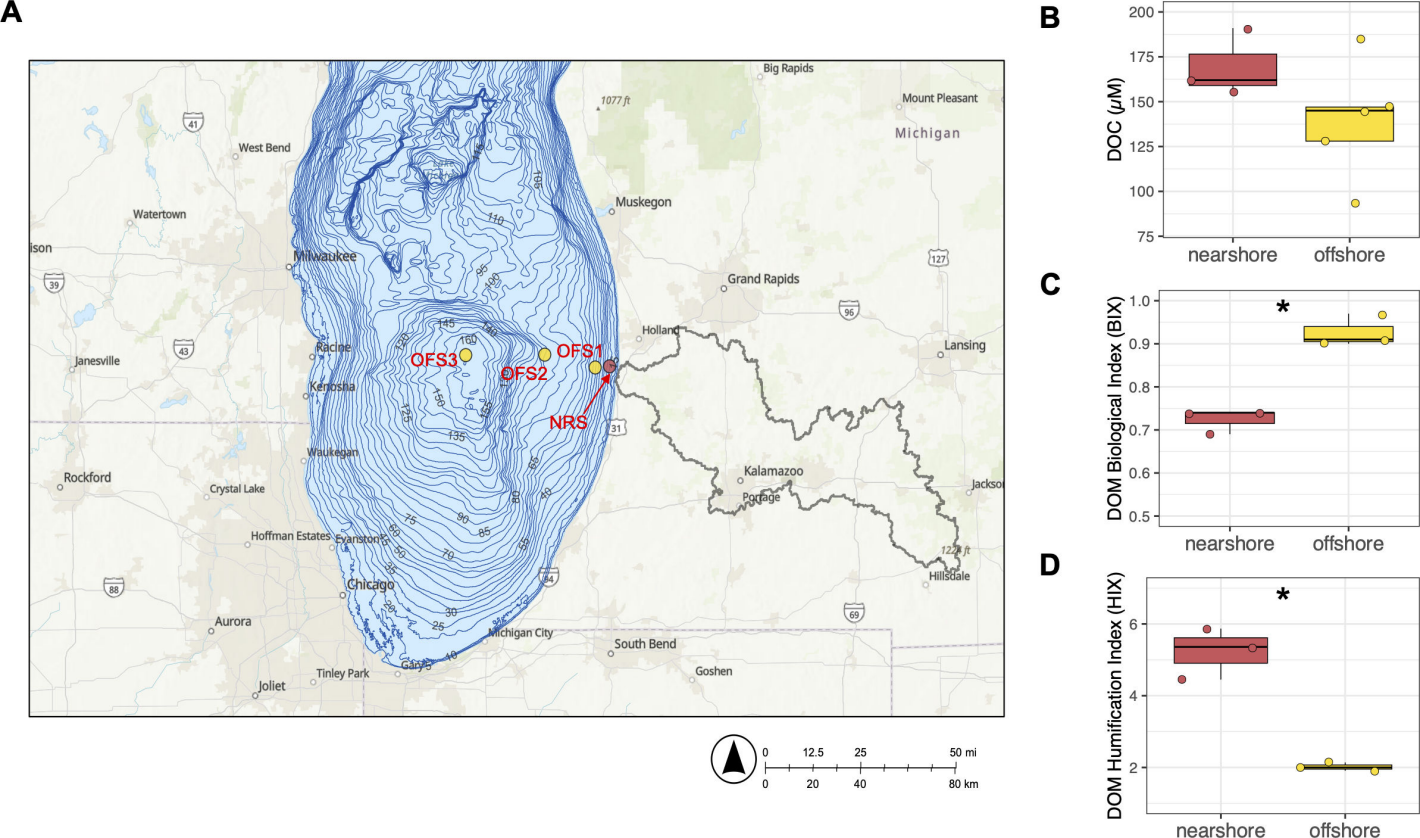
(**A**) Map of southern Lake Michigan with the nearshore (NRS) and offshore (OFS) sampling sites labeled and lake bathymetry information included. The Kalamazoo River watershed is also outlined in the map in black. The map was created using ArcGIS. (**B**) Dissolved organic carbon concentrations in nearshore and offshore Lake Michigan from 2017 to 2018 cruises. (**C and D**) Dissolved organic matter characteristics based on biological index (BIX) and humification index (HIX) as derived from fluorescent EEM spectra of DOM from nearshore and offshore Lake Michigan surface water. The DOC/DOM parameters that were significantly different between nearshore and offshore are highlighted by a ‘*’ symbol in the panel (Welch’s *T* test, *P* < 0.05).

Lake Michigan is a large, dimictic freshwater lake located in the United States. Near-surface (0–2 m depth) Lake Michigan water samples were collected across a nearshore-to-offshore transect beginning near the mouth of the Kalamazoo River, one of the largest tributaries to southern Lake Michigan (19). The transect lies in the middle of southern Lake Michigan, with the coastal regions of the transect likely subject to alongshore currents that are part of the gyre-like circulation pattern in southern Lake Michigan (8). Samples were collected in summer and spring seasons during 2017–2018 from four sites across the transect: three samples from nearshore site NRS (~3.5 km from shore), and a total of 8 samples from offshore sites OFS1, OFS2, and OFS3 (Fig. 1A). The offshore sites OFS1, OFS2, and OFS3 correspond to the stations MI48b, MI19, and MI18, respectively, that are part of the annual spring and summer survey efforts by the U.S. Environmental Protection Agency (EPA). More details about the collected samples are provided in Table 1.

**TABLE 1:** Sample metadata for Lake Michigan surface water and associated nutrient measurements (soluble reactive phosphate; nitrate+nitrite; dissolved organic carbon)

Sample site	Sample type	Latitude, longitude	Sampling date	Distance from shore (km)	Soluble reactive phosphate (SRP) μg P/L	NO_x_ μg N/L	DOC±SD (μM)
NRS	Nearshore	42.693056, –86.258333	8/8/17	3.5	1.3	290	191 ± 1.2
NRS	Nearshore	42.693056, –86.258333	4/8/18	3.5	2.9	505	162 ± 0.9
NRS	Nearshore	42.693056, –86.258333	5/27/18	3.5			156
OFS1	Offshore	42.689444, –86.331944	8/3/17	10	2	258	185 ± 0.9
OFS1	Offshore	42.689444, –86.331944	3/29/18	10	2	268	128 ± 0.5
OFS1	Offshore	42.689444, –86.331944	8/2/18	10	3.2	344	
OFS2	Offshore	42.734167, –86.592222	8/3/17	30	2.5	293	145 ± 0.4
OFS2	Offshore	42.734167,–86.592222	3/29/18	30	1.9	333	147 ± 1.1
OFS2	Offshore	42.734167, –86.592222	8/2/18	30			
OFS3	Offshore	42.7325, –86.999167	3/29/18	50	1.8	365	93 ± 1.4
OFS3	Offshore	42.7325, –86.999167	8/2/18	50	2.4	314	

Except for the NRS sample from May 2018, all other samples were collected in the night after sunset to avoid the confounding effects of diel variation on microbial community function. Distinct metabolic functions tend to predominate in the day (e.g., photosynthesis and oxidative phosphorylation) versus the night (e.g., sugar uptake/transport) for the microbial populations in lake ecosystems (30, 31). Offshore water samples were collected onboard the research vessel *R/V* Lake Guardian during EPA’s spring and summer Great Lakes surveys. For nearshore, water was collected using the horizontal sampler (Wildco, Yulee, FL) on small boats. For each sampling event, between 4 and 10 L of water were immediately filtered through pre-combusted 1.6-µm pore-size glass fiber filters (Sterlitech, Kent, WA) to remove larger particles and organisms, and free-living cells were collected on 0.2-µm pore-size polycarbonate membrane filters (EMD Millipore, Billerica, MA). All the filter tubing and polycarbonate carboys/cubitainers used in sample collection and processing were acid-cleaned with 10% vol/vol hydrochloric acid, followed by rinsing with deionized water. Filters were stored immediately either in liquid nitrogen or dry ice and transported back to the lab for storage at −80°C until further processing. The total processing time between water collection and filter storage in liquid nitrogen ranged between 40 and 90 min for all the samples. In addition, about 100 mL of each sample filtrate through the 0.2-µm pore-size filters was collected in pre-combusted scint vials for use in nutrient/DOC measurements and DOM characterization. Roughly half of the filtrate was stored frozen at −20°C, and the remaining filtrate was stored at 4°C in aluminum foil-covered scint vials. Lastly, in addition to the above-described sampling design, we were able to obtain 2 ~1 L water samples from the nearshore NRS site in summer 2018. These samples were stored at 4°C for 1–2 weeks prior to transport to the laboratory for analysis. We utilized these water samples for DOM characterization only.

### Nutrients, DOC measurements, and DOM characterization

For all the samples, part of the stored frozen filtrate was used for measuring nutrients (PO_4_^3-^, NO_x_^-^) at the University of Illinois, Chicago, using an autoanalyzer (AQ300, SEAL Analytical, Mequon, WI) (32). The remaining frozen filtrate was used for measuring DOC concentration at the School of Freshwater Sciences, University of Wisconsin-Milwaukee, using the high-temperature combustion method (25). Filtrate stored at 4°C for each sample was used for characterizing the chromophoric DOM (CDOM) and fluorescent DOM (FDOM) using methods and techniques as described in a previous study (25).

For CDOM/FDOM analysis, we were able to store and process filtrate for all the spring 2018 samples and some samples from summer 2018; thus, we only present CDOM/FDOM results here for this partial data set. For CDOM, UV-vis absorption spectra scanned in the 200–1,100 nm range were used to calculate optical parameters, including the absorption coefficient at 254 nm (a_254_, in m^−1^); spectral slope over the wavelength range of 275–295 nm (S_275-295_, in nm^−1^); and slope ratio, Sr (ratio between linearly fitted S_275–295_ and S_350–400_) (25). Briefly, a_254_ serves as a proxy for the overall concentration of CDOM, reflecting the abundance of irradiance-absorbing organic compounds. Values of S_275–295_ and S_r_ are inversely correlated to DOM molecular weight (33), with higher S_275–295_ or S_r_ values indicating a predominance of low-molecular-weight DOM molecules and lower values associated with high-molecular-weight bio-molecules, often of labile or freshly produced origin (34).

For FDOM, EEM spectra measured on the spectrofluorometer were used to derive optical properties, including biological index (BIX) and humification index (HIX), and to identify major fluorescence peaks and/or components (35). These fluorescence indices, BIX and HIX, were used to indicate the freshness and humification level of DOM. Specifically, BIX was calculated as the ratio of fluorescence intensity at 380 nm over that at 430 nm under the excitation wavelength of 310 nm (36). In general, BIX ranges from 0.4 to 1.0 in freshwater ecosystems (37), with higher BIX values (> 0.7) indicating the dominance of autochthonous DOM. HIX was derived from the ratio of fluorescence signals over the emission range of 435–480 nm to those over the range of 300–345 nm with excitation at 254 nm (38), with values normally ranging between 0 and 10 depending on the contribution of terrigenous DOM.

### RNA isolation and sequencing

RNA was isolated from frozen 0.2-µm pore-size filters using an organic extraction method as described previously (22). Briefly, filter fragments were first incubated in a lysis buffer (50 mM Tris-HCl, 40 mM EDTA, and 0.75 M sucrose) with 1.15 mg/mL lysozyme at 37°C for 30 min, followed by incubation with 1% SDS and 10 mg/mL proteinase K at 55°C for 2 h while rotating. RNA was extracted from the lysate using acid phenol:chloroform (pH 4.5), followed by isolation by ethanol precipitation and subsequent elution in RNase-free water. Isolated RNA was then treated with TURBO DNA-free DNase kit (Invitrogen, Carlsbad, California) to digest residual genomic DNA, followed by cleanup and concentration using ethanol precipitation and elution in RNase-free water. The cleaned, concentrated total RNA was then submitted to the University of Illinois Chicago’s Genomics Research Core (GRC) for metatranscriptome library preparation and sequencing. Library preparation first involved enrichment of the mRNA pool using Lexogen’s RiboCop rRNA Depletion kit for Bacteria, followed by library preparation using CORALL Total RNA-Seq Library Prep kit. Metatranscriptome libraries were sequenced on Illumina’s NovaSeq S4 platform in paired-end format and 150 bp read length, yielding between 43.8 and 58.6 million paired-end reads per library. Further information about sequencing yield, sequences retained after quality trimming, and ribosomal RNA read removal is provided in Table S1.

In addition to the metatranscriptome sequencing described above, a portion of filter fragments for samples from summer 2017 was used for isolation of community genomic DNA (gDNA) using the same organic extraction method as described above (with the modification of including RNase in the lysis step and using phenol:chloroform pH 8.05) (39). Isolated gDNA for 2017 samples was then submitted to GRC for metagenomic sequencing using Illumina’s NextSeq platform in paired-end format and 150 bp read length.

### Bioinformatics analysis of sequencing data

The metatranscriptomes (MTs) were first processed for adapter removal using Trimmomatic v0.33 (40), followed by read trimming with an in-house script using a minimum Phred score of 20 across a 4-base sliding window and not allowing any N-base calls. The trimmed MT libraries were first individually assembled using Megahit v1.1.1-2 (41), and the assemblies were subsequently submitted to the Integrated Microbial Genomes & Microbiomes (IMG/M) system (IMGAP v5.1.17) for gene prediction and annotation (42). IMG/M utilized GeneMark.hmm-2 v1.25 and Prodigal v2.6.3 for predicting coding sequences. The predicted coding sequences were functionally annotated using the following databases: COG 2014, TIGRFAM 15.0, SuperFamily 1.75, Pfam 34.0, Cath-Funfam 4.2.0, SMART 01_06_2016, Rfam 13.0, and KEGG.

All short-read MT libraries were processed through SortMeRNA to predict ribosomal RNA (rRNA) reads in the data (43). Subsequently, the non-rRNA reads from each MT library were mapped to the corresponding predicted coding sequences from IMG/M using blastn with the following filtering parameters: 98% sequence identity, 70% query coverage, evalue < 10^−20^, and only retaining the best hit for each query. The mapping results were then used for calculating the transcript abundance for each predicted coding sequence using the *BlastTab.seqdepth_nomedian.pl* script from the Enveomics toolbox (44). The transcript counts for all the coding sequences annotated within a specific gene family were summed for each MT library, and the results for all the libraries were merged into a single feature abundance matrix for the gene families. A filtering threshold was applied to the matrix, where only those features with at least 10 transcript counts across at least 30% of the samples (4 out of the 11) were retained. The filtered feature table retained 5,316 unique gene families, which were first normalized for different library sizes using size-factor normalization in DESeq2.0 (45).

### Statistical analysis and data visualization

All statistics and data visualizations were performed in R v.4.3.2, unless noted otherwise. The DESeq2.0-normalized gene family feature table was utilized for calculating pairwise Bray-Curtis distances between samples using the vegan v2.6-10 package in R (https://github.com/vegandevs/vegan). The pairwise distances were then utilized for principal coordinate analysis (PCoA) using the ecodist v2.1.3 R package (46), followed by visualization of samples in the ordination space using ggplot2 v3.5.2 (https://ggplot2.tidyverse.org/). The normalized feature table was also utilized in a mixed-effects modeling approach to detect potential gene families associated with variables of interest using MaAsLin2 v1.16.0 (47). For the modeling, “location” (nearshore/offshore, with nearshore as reference) and “season” (spring/summer, with spring as reference) were used as fixed effects, and sampling station (OFS1/OFS2/OFS3/NRS) and sampling year (2017/2018) were used as random effects. We set the analysis method to “NEGBIN” (negative binomial), with no data transformation or normalization. Finally, we applied false discovery rate correction to the model results using the Benjamini-Hochberg method, with a significance threshold (q-value) set at 0.05.

In addition to the statistical analysis of all the gene families described above, we also performed a focused analysis of transporter gene expression for C, N, and P substrates (Fig. 4). For this, we first mined for transporter gene features from the full feature table by searching for the following keywords in feature names in an iterative and hierarchical manner: “transport,” “permease,” “ABC,” “TonB,” “uptake,” and “porin.” To the resulting list, we then manually assigned broader substrate categories to the individual gene families based on information available on reference databases (KEGG/COG) and literature review. This gene family-to-broad substrate mapping file for C, N, and P substrates is available as Table S2. Finally, gene expression counts were then summed for each of the broad substrate categories and utilized for mixed-effects modeling in the same way using MaAsLin2 as described above for the full feature table.

### Taxonomic and population-specific analysis of metatranscriptomes

To characterize the taxonomic composition of the active (mRNA-based) bacterial communities in Lake Michigan, we utilized the MTs in combination with the metagenomes generated from summer 2017. These metagenomes (MGs) were first individually assembled using MEGAHIT v1.1.1-2. Assembled contigs > 500 bp for each metagenomic library were then mined for protein-coding genes using MetaGeneMark (48). The predicted genes and their contigs were then utilized for taxonomic classification using MyTaxa (49) and its database of bacterial and archaeal genomes (http://enve-omics.ce.gatech.edu/data/mytaxa). MyTaxa employs a combination of approaches to classify assembled metagenomic contigs, including homology-based classification of predicted genes for a contig and identifying their frequency of horizontal gene transfer. We then mapped the rRNA-filtered transcript reads for each sample to the annotated genes using blastn, followed by coverage calculation for each gene using the *BlastTab.seqdepth_nomedian.pl* script from Enveomics. MT samples from a specific location were only mapped to the annotated genes from the corresponding MG, with the exception of MTs for site OFS3 that were mapped to the genes corresponding to site OFS2. Finally, to identify coverage trends for specific microbial populations across Lake Michigan, we mapped the transcript reads to Metagenome Assembled Genomes (MAGs) generated from Lake Michigan in our prior work (50) (Chapters 3 and 4). Metatranscriptomic reads were mapped to the MAGs similarly as described above for the assembled genes, followed by visualization of the mapping parameters (percent identity of alignment and coverage) using read recruitment plots from the *enveomics.R* package in R.

## RESULTS

### Nutrients and DOC/DOM characteristics across southern Lake Michigan

Nearshore and offshore Lake Michigan waters did not significantly differ in their nutrient and DOC concentrations (Table 1; Fig. 1B). Specifically, there were no significant differences in average phosphate and nitrate levels between nearshore and offshore (Welch’s *t* test, *P* = 0.88 and *P* = 0.57, respectively). DOC concentrations were also not significantly different between the two regions (Welch’s *t* test, *P* = 0.15), although the mean DOC concentration in nearshore (170 µM, *n* = 3) was 21.6% higher than in offshore (140 µM, *n* = 5) (Table 1; Fig. 1B). Finally, we also calculated nutrient ratios for the major nutrients measured in this study, that is, DOC:NO_x_; DOC:SRP; and NO_x_:SRP. Comparison of these nutrient ratios between nearshore and offshore waters did not reveal any significant differences (Welch’s *t* test, *P* > 0.05).

Chromophoric and fluorescent DOM characteristics displayed strong patterns of differences between nearshore and offshore Lake Michigan (Fig. S1; Table 2). Optical properties for characterizing CDOM, such as the absorption coefficient (a_254_, a proxy of bulk DOC concentration), spectral slope (S_275-295_), and slope ratio (S_R_), which both are indicators of apparent DOM molecular weight through an inverse correlation, were all significantly different between the nearshore and offshore samples (Welch’s *t* test, *P* ≤ 0.05). We saw higher a_254_, lower S_275–295_, and lower S_R_ values for nearshore CDOM as compared to offshore. From the fluorescence EEM spectra, we found the presence of signatures of humic-like DOM (peak A and C) and protein-like DOM (peak B) in the samples (Fig. S1). However, there were differences in the relative intensity of peaks B and C between the nearshore and offshore samples. Nearshore samples seemed to have a more pronounced peak C, which has been associated with terrestrial humic-like DOM (25, 51). Conversely, offshore samples had a relatively more pronounced peak B, which has been associated with autochthonous DOM.

**TABLE 2:** Dissolved organic matter characteristics across southern Lake Michigan in 2018

Sample site	NRS	NRS	NRS	OFS1	OFS2	OFS3
Sample type	Nearshore	Nearshore	Nearshore	Offshore	Offshore	Offshore
Season	Spring	Summer	Summer	Spring	Spring	Spring
DOC (μM)	162			128	147	93
Absorption coefficient at 254 nm, a254 (m^−1^)	8.73	7.71	9.34	4.27	4.01	4.11
S_275-295_ (nm^−1^)	0.022	0.023	0.023	0.03	0.031	0.029
S_R_	1.409	1.461	1.274	1.956	2.235	2.767
BIX	0.69	0.74	0.74	0.9	0.91	0.97
HIX	5.36	4.45	5.87	2.14	2	1.91

The values of biological index (BIX, a DOM source indicator) and humification index (HIX, an indicator of DOM humification extent) derived from the EEM data complemented the trends from Fig. S1: nearshore DOM had significantly lower mean BIX than offshore DOM (Welch’s *t* test, *P* < 0.05), suggesting lower proportion of biogenic DOM in nearshore water (Fig. 1C), and the mean HIX of the nearshore samples was significantly higher than the offshore (Welch’s *t* test, *P* < 0.05) (Fig. 1D), indicating higher terrigenous DOM in nearshore water (35).

### Bacterial community dynamics in southern Lake Michigan

More than 85% of the metatranscriptomic sequences analyzed by MyTaxa across the samples were classifiable at the phylum level, with only ~40% of the sequences on average classified at the lower taxonomic levels. We focused subsequent taxonomic analysis at the phylum level (with Proteobacteria subdivided into classes). The phylum-level mRNA-based active bacterial community composition was largely similar in nearshore and offshore Lake Michigan (Fig. S2). Sample type: nearshore/offshore (or distance from shore), season, and sampling year did not significantly explain the variation in bacterial community composition (PERMANOVA, *P* > 0.05, Bray-Curtis metric). Bacterial communities were primarily comprised of phyla (and subphyla) Actinobacteria, Betaproteobacteria, Alphaproteobacteria, unclassified Proteobacteria, and Bacteroidetes (Fig. S2). Other taxa included Verrucomicrobia, Cyanobacteria, Gammaproteobacteria, and Deltaproteobacteria. Notably, Betaproteobacteria had a higher mean relative abundance in mid-offshore site OFS1 (16.8%) than in nearshore (4.29%) or further offshore sites OFS2 (6.42%) and OFS3 (5.63%).

To evaluate trends for specific microbial populations across Lake Michigan, we mapped the MTs to MAGs generated from multiple metagenomic data sets from this transect (50) (Chapters 3 and 4). We focused our efforts on seven MAG-based populations representing some of the abundant bacterial lineages in Lake Michigan (Table S3). For three of these MAGs (bin004, bin009, and bin010), we observed roughly similar coverage and distribution across the nearshore and offshore MTs. These included the MAGs bin004 and bin009 classified as *Limnohabitans* (Fig. S3) and *Polynucleobacter* (Fig. S4), respectively. These are both abundant, widely distributed freshwater lineages of Betaproteobacteria (52). We observed a roughly consistent distribution for MAG bin040 (AcI-B1 Actinobacteria) across space and time; however, the MAG had low sequence identity with the recruited cDNA reads (Fig. S5). The other 3 MAGs exhibited more variable distribution in transcript abundance across the samples. These included MAGs classified as *Synechococcaceae* (bin035) and *Fluviicola* (bin181). *Synechococcaceae* bin035 was mainly abundant in summer 2017 and at the far offshore sites OFS2 and OFS3 (Fig. S6), whereas *Fluviicola* bin181 exhibited sporadic presence primarily in the nearshore and the closest offshore site OFS1 (Fig. S7).

### Bacterioplankton gene expression for DOM metabolism differs in nearshore and offshore Lake Michigan

**Fig 2:**
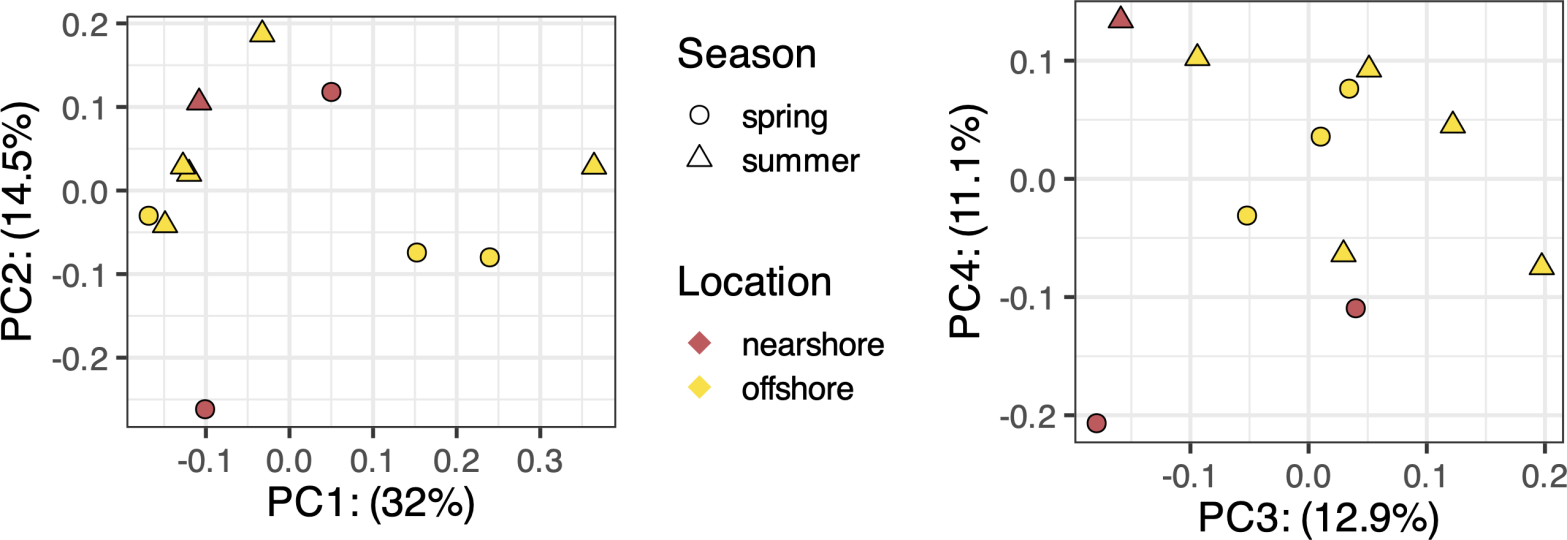
Principal coordinates analysis (PCoA) plot (based on Bray-Curtis distance metric) of functionally annotated and DESeq2-normalized metatranscriptomes for Lake Michigan microbial communities 2017–2018. The left panel shows sample positions in the ordination space for the first two principal coordinates, and the right panel shows sample positions in the ordination space for principal coordinates 3 and 4.

**Fig 3:**
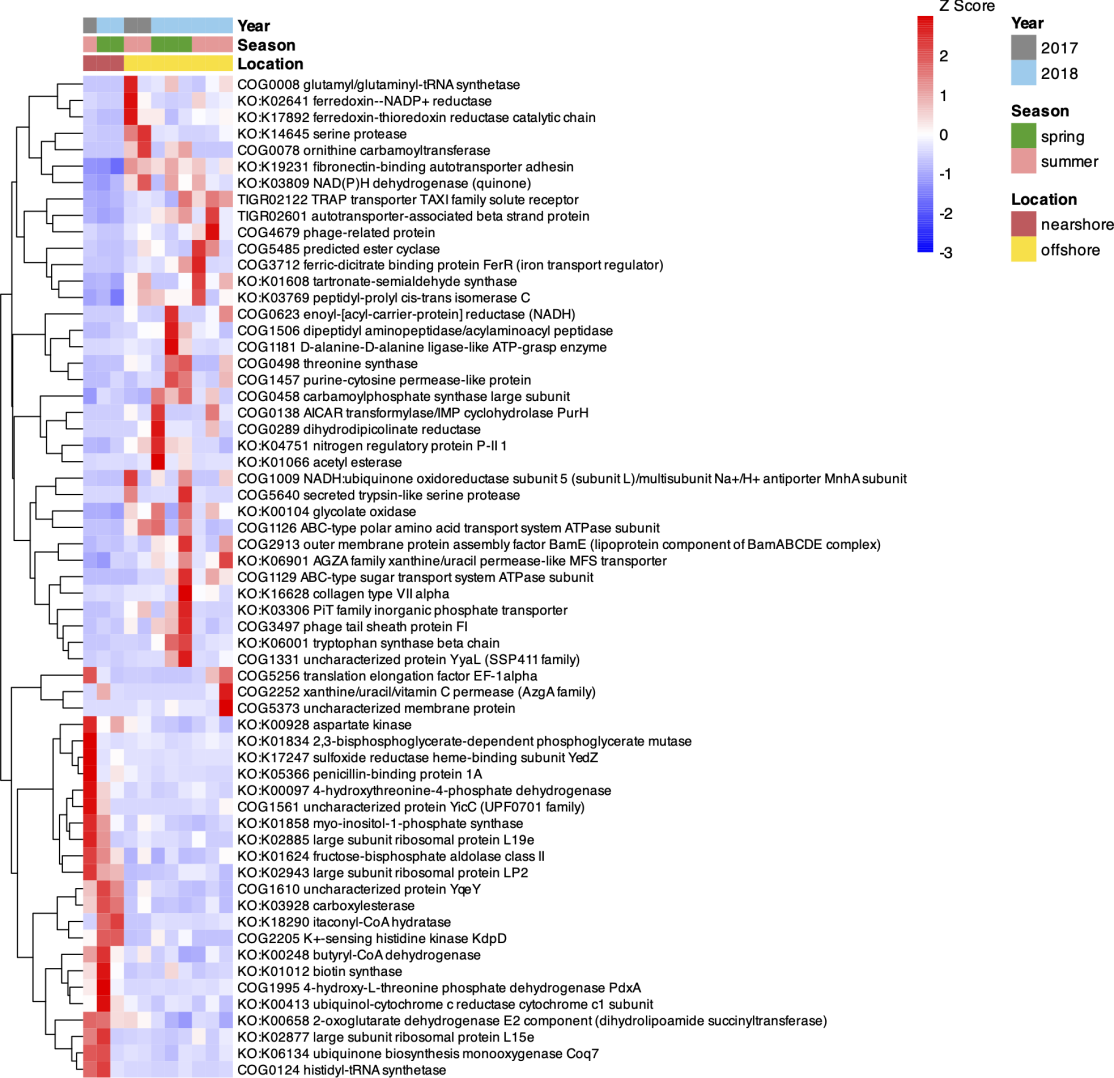
Heatmap of DESeq2-normalized and Z-score transformed expression levels for gene families with significantly different expression between nearshore and offshore Lake Michigan (MaAsLin2-based mixed-effects modeling, FDR-corrected q-value < 0.05). The heatmap shows the top 60 most significant features based on q-value (full list of gene families provided in Table S4).

**Fig 4:**
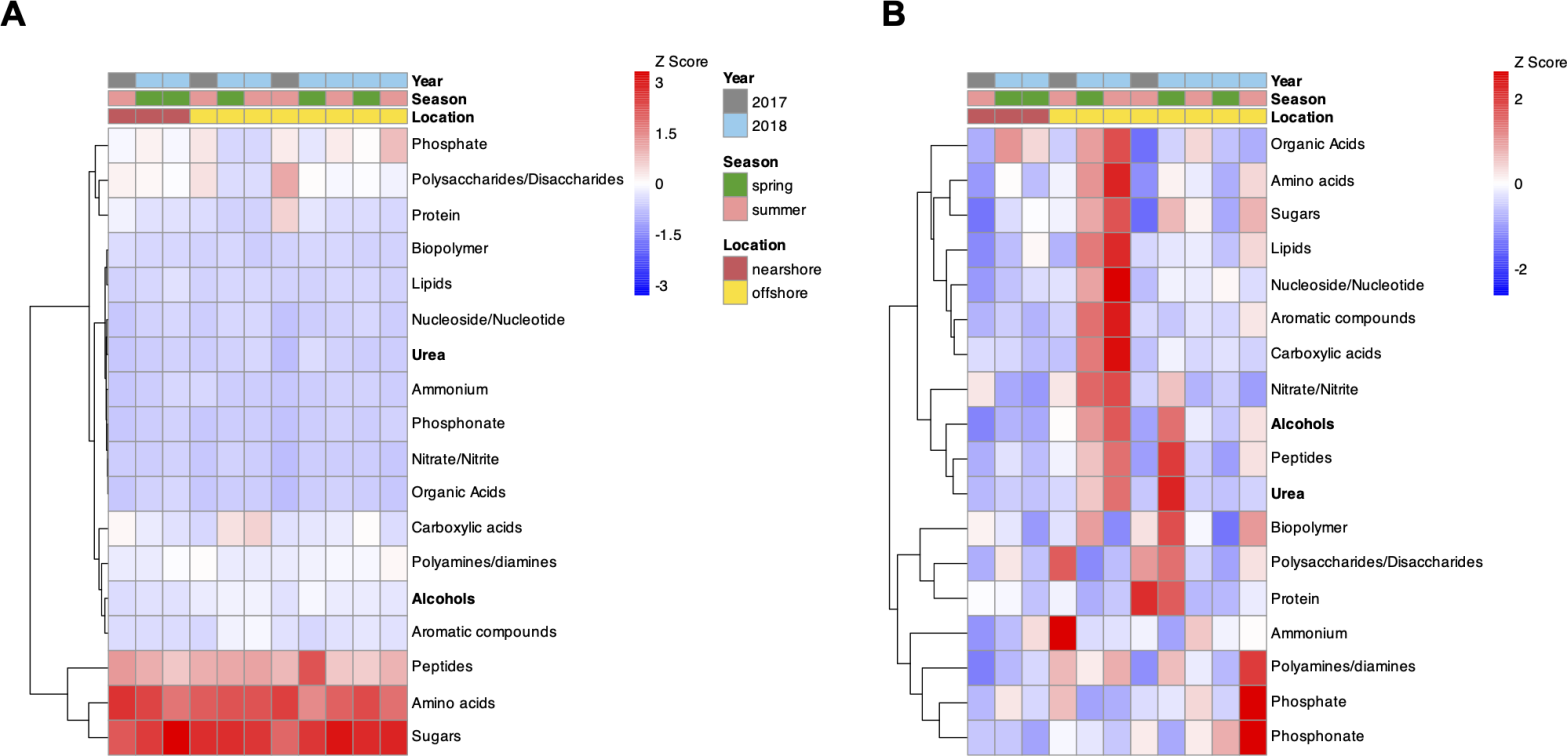
Heatmaps representing gene expression levels for transporter genes for different carbon, nitrogen, and phosphorus substrates in Lake Michigan bacterioplankton. Gene expression levels were normalized to account for different library sizes for each sample using size-factor normalization in DESeq2. The heatmap in panel (**A**) represents gene expression levels standardized using Z score across each sample to focus on within-sample contrast in gene expression for all the substrates, whereas panel (**B**) represents gene expression levels standardized with Z score across each transporter gene category to focus on between-sample contrast. Transporter gene categories with significantly different gene expression between nearshore and offshore Lake Michigan bacterioplankton are highlighted in bold font (MaAsLin2-based mixed-effects modeling, q-value < 0.05).

The overall gene expression profile of the bacterial communities did not exhibit notable clustering based on site or season (Fig. 2). However, gene expression for several gene families showed significant association with location or season (total *n* = 310). Particularly, 130 gene families had significant differential expression between nearshore and offshore bacterial communities (MaAsLin2-based mixed effects modeling, FDR-adjusted q-value < 0.05) (Fig. 3; Table S4). Of the 130 differentially expressed gene families, we focused further analysis on genes with potential relevance for major nutrients/substrate (C, N, and P) acquisition and associated metabolism. This included transporter genes associated as markers of cellular C (high-affinity sugar/organic acid transporters) and N (ammonium, nitrate/nitrite, and urea transport) stress. We also examined the gene expression patterns for all the C/N/P substrate-specific transporter genes in more detail below (Fig. 4). Offshore bacterial communities exhibited higher expression of several gene families involved in the uptake and degradation of DOM substrates that included peptidases, proteases, lyases, and transporter genes for amino acids, nucleobases, sugars, and sugar alcohols (Fig. 3; Table S4). In contrast, there were significantly fewer gene families related to DOM substrate acquisition and metabolism that had more transcripts in the nearshore (Fig. 3). These gene families included fructose bisphosphate aldolase, carboxylesterase, and inositol oxygenase. In addition, certain gene families related to cellular housekeeping functions and energy metabolism were higher in expression in the nearshore bacterioplankton, such as ribosomal protein biosynthesis, ubiquinone biosynthesis monooxygenase, and ubiquinol cytochrome c reductase (Fig. 3; Table S4). Finally, offshore bacterial communities also exhibited more transcripts for gene families involved in the biosynthesis of certain amino acids, inorganic phosphate transport, and nitrogen regulatory protein (Fig. 3; Table S4). In addition to the transporter gene analysis described below, we mined the MaAsLin2-based results for other gene families known for being potential markers of C/N/P cellular stress in prokaryotes (Table S5). These included phosphorus-stress markers (regulators such as phoR–phoB, phosphatases including phoA, phoD, and phoX, phosphonate catabolism genes, lipid remodeling pathways, and polyphosphate metabolism), nitrogen-stress markers (e.g., glutamine synthetase and nitrogenases), and carbon-stress markers (carbon-concentrating mechanisms and glyoxylate shunt genes). We did not find any of these stress-marker genes among the differentially expressed genes across the transect (Table S4).

Transporter gene expression for substrates can serve as an indicator of nutrient/substrate demand in the microbial community as well as substrate availability in the environment (53). We performed a focused comparative analysis in the gene expression profile of all C, N, and P transporter gene systems between the nearshore and offshore bacterial communities (Fig. 4). The results demonstrated a similar substrate affinity among the transporter genes between nearshore and offshore (Fig. 4A). However, the offshore bacteria had an overall higher expression for these transporter genes, including higher expression of transporter genes for substrates alcohols and urea (MaAsLin2-based mixed effects modeling, FDR adjusted q-value < 0.05) (Fig. 4B).

## DISCUSSION

In this study, we monitored the bacterial community activity across a spatial environmental gradient in Lake Michigan using a metatranscriptomics approach. Metatranscriptomics data were analyzed in a rich environmental context provided by nutrient measurements and spectrofluorometric characterization of the bulk DOM pool. This approach enabled us to systematically investigate bacteria-water chemistry relationships in one of the largest freshwater ecosystems in the world. Prior research in Lake Michigan has characterized bacterial ecophysiology in terms of abundance, secondary production, respiration, growth, community structure, and activity in coastal and offshore regions (14, 18–22). These studies have elucidated the importance of phytoplankton-derived DOM as the dominant factor driving bacterial production in Lake Michigan, and a smaller but significant contribution coming from terrigenous sources such as tributary inputs.

The water chemistry results highlighted similarities in dissolved inorganic N and P (NOx and SRP) levels between nearshore and offshore regions, and DOC had a minor drop in concentration in the offshore. We also did not observe significant differences in the nutrient ratios between the two regions. However, the two regions showed significant differences in their DOM characteristics. Significant differences in the CDOM characteristics (a_254_, spectral slope [S_275–295_], and slope ratio [S_R_]) suggested that a larger proportion of nearshore DOM comprised of higher molecular weight and potentially labile components (25, 34). Similarly, a more pronounced peak C in the FDOM EEM spectra for nearshore provides evidence for a greater presence of humic-like DOM that is likely derived from terrigenous sources (25, 51). A higher HIX for nearshore FDOM provides further evidence of a more pronounced presence of humic compounds in the nearshore. These results are not surprising, given the proximity of nearshore regions in southeastern Lake Michigan to large tributaries such as the Kalamazoo River with a watershed of 5,200 km^2^ (Fig. 1). Moreover, alongshore currents along the southeastern coast of the lake that are part of the gyre-like cyclonic circulation pattern in southern Lake Michigan initially limit the mixing of nearshore-offshore waters (8, 28, 29), likely affecting the rate of transport of terrigenous DOM to offshore. In contrast, offshore waters comprised a higher proportion of DOM produced from autochthonous sources (higher BIX than nearshore) and with a relatively higher fraction as protein-like components, such as peak B in the EEM spectra. As DOC slightly declined from nearshore to offshore, the higher terrestrial DOM (tDOM) in nearshore waters might be contributing to the elevated DOC levels. Overall, these results imply that differences in DOM quality, rather than major changes in nutrient (C/N/P) concentrations, are the major environmental gradient along the coastal-to-offshore transect in southern Lake Michigan.

The mRNA-based taxonomic community composition was largely similar across Lake Michigan. Similar to what has been observed in the 16S ribosomal RNA gene amplicon-based studies for Lake Michigan (14, 21), the major active taxa observed in this study included Actinobacteria, Bacteroidetes, Betaproteobacteria, and Alphaproteobacteria. Cyanobacteria were abundant in the offshore in summer 2017, as seen before in the mRNA-based community profiles (22), but we did not observe similar high abundances in 2018. The patchy distribution of *Fluviicola* MAG bin181 might be associated with pulses of phytoplankton-derived labile DOC, as *Fluviicola* are known to be rapid consumers of phytoplankton-derived DOC (54). Finally, the lower sequence identity between the AcI-B1 MAG and the recruited cDNA reads likely signifies the presence of closely related, but divergent populations in the samples to this MAG (Fig. S5). The presence of active but divergent AcI populations in Lake Michigan is interesting. Given the diversity in substrate preferences within this group, this result might reflect substrate diversity in the lake DOM pool (55).

Comparison of gene expression between nearshore and offshore bacterioplankton revealed significantly higher expression in the offshore for several genes related to the uptake and degradation of DOM substrates (Fig. 3 and 4). We also observed higher gene expression for one inorganic phosphate transporter gene family in the offshore and for gene families associated with urea transport. Elevated expression of transporter genes and enzymes for the acquisition of specific nutrients generally indicates nutrient limitation and demand in the bacterial population/community (11, 56). The gene expression results observed here overlap with the significant differences observed in the DOM characteristics across Lake Michigan. Significant correlation between DOM molecular composition and microbial diversity was also observed in temperate lakes in Croatia (7), although there were also distinct factors affecting DOM and microbial community assembly separately. Similarly, incubation experiments with soil and surface water bacterial communities highlighted the significant influence of DOM variation on bacterial community composition and carbon processing (4). Studies in marine coastal to offshore transects have also found strong correlations between the active microbial community and DOM molecular composition (9). Overall, the results in our study suggest that there is greater metabolic investment in (predominantly) organic substrate acquisition in offshore bacterioplankton to satisfy C (and perhaps P and N) demands and that this could be associated with the lower bioavailability of the offshore DOM pool. There is evidence to suggest that a significant component of the pelagic DOM pool in Lake Michigan may be biologically reworked and thus recalcitrant (15, 25). In addition, Lin and Guo observed significantly high molar DOC/DOP ratios (> 600) in offshore Lake Michigan in 2013–2014 (25, 57), indicative of a P-limited DOM pool in the offshore that might affect bacterial metabolism. Finally, as we primarily found evidence for differential expression of markers for cellular C stress (e.g., sugar transporters) and less so for N and P stress markers (Table S5) across the transect, we speculate that the offshore bacterial communities might be investing in DOM acquisition to satisfy cellular C demand.

In contrast, few gene families showed higher gene expression for DOM acquisition in the nearshore Lake Michigan bacteria. Additionally, we observed higher nearshore gene expression for housekeeping functions and energy metabolism. Nearshore bacterioplankton have been documented to exhibit higher secondary production (18) and respiration (20) in comparison to offshore in the past. The gene expression patterns in our study likely provide molecular-level evidence for the previous bacterial physiological data for a more productive nearshore region in Lake Michigan. Higher phytoplankton and chlorophyll *a* levels observed in the nearshore waters, particularly in the spring season, potentially imply that autochthonous production can be an important source of labile DOM substrates for nearshore bacterioplankton (58, 59). In their study, Pothoven and Vanderploeg conclude that the invasive dreissenid mussel proliferation in Lake Michigan seems to have had a stronger negative effect on the offshore chlorophyll *a* than in nearshore areas, where tributary loadings seemed to have offset some of the mussel filtering effects on the chlorophyll *a* and total phosphorus levels (59). In addition, the higher tDOM in the nearshore might be satisfying some of the nearshore bacterial substrate demand. The northward alongshore currents along the southeast coast of the lake might also initially inhibit the transport of tDOM and terrestrially derived nutrients to deeper offshore waters (8, 28). There are various mechanisms through which the tDOM might become bioavailable to lake bacteria. Photochemical alteration of tDOM due to enhanced sunlight exposure in lake waters might increase its bioavailability for bacterial production (60). Moreover, tDOM bioavailability can also be enhanced in the presence of phytoplankton-derived labile carbon, based on the priming hypothesis (61). In our previous study, we saw preliminary evidence at the transcription level for the capacity of Lake Michigan bacterioplankton to metabolize allochthonous DOM (22).

Despite the differences in the expression of DOM acquisition genes, the similarity in C, N, and P substrate preferences between the nearshore and offshore bacterioplankton is interesting. The major substrates included simple sugars, amino acids, peptides, carboxylic acids, polysaccharides, and inorganic phosphate (Fig. 4). Many of these organic compound classes have been observed as important substrates for bacteria in other lakes and aquatic ecosystems (53, 62, 63). Work done in Lake Zurich has provided valuable information about substrate preferences and niche specialization in bacterioplankton populations (64). However, a similar assessment of bacterial substrate preferences in the large lakes like Michigan is lagging, although they contain 15% of the global lake-water DOC (15). To our knowledge, this is the first study that has characterized the DOM substrate preferences for Lake Michigan bacterial community at a molecular level. Peptides, carbohydrates, amino acids, and carboxylic acids have been shown to be associated with the lysate and exudate of phytoplankton cells (53, 65, 66). Carboxylic acids can also be produced from the photochemical degradation of terrestrially derived humic DOM when exposed to solar radiation in lake waters (67). Overall, the relative importance of the different sources of DOM substrates observed here for Lake Michigan bacteria supports what we know about DOM sources and bacterial physiology from prior studies (19): phytoplankton-derived DOM is still the dominant source of labile substrates for Lake Michigan bacterioplankton, with terrestrial DOM also playing a smaller but significant role.

There are a few limitations associated with this study. First, the lack of molecular resolution of DOM characterization limits the scope of interpretations that can be made. Although the spectrofluorometric DOM characterization performed here provides valuable information about the lake water chemistry, recent use of mass spectrometric approaches such as FT-ICR-MS to study freshwater DOM has enabled a more robust characterization of DOM molecular composition at a similar level of resolution as microbial species/OTUs (7). Additionally, caution should be exercised in the interpretation of metatranscriptomics data, as gene copy number variation across taxa can influence the overall community expression profile and confound the actual taxonomic composition. Another important limitation of utilizing metatranscriptomics is the relative nature of the data; in the absence of any quantitative approaches (e.g., RT-qPCR/ddPCR on universal markers or the use of External RNA Controls Consortium (ERCC) spike-ins with metatranscriptomics), we cannot compare the absolute number of transcripts across the samples. Finally, another potential limitation of our study could be the lower temporal resolution of the data. Although large lakes are relatively more stable in their ecological profile compared with smaller freshwater ecosystems, our sampling scheme likely did not capture the full scope of variation in microbial and water chemistry dynamics in Lake Michigan. Surveys of large lakes are also complicated due to limited access to pelagic waters, as they require coordinated sampling efforts with scientific research vessels. Nonetheless, future research efforts utilizing more sophisticated water chemistry profiling over a more temporally resolved scale can improve/verify the conclusions made in this study.

In conclusion, the results from this study imply that the bacterial communities in different regions of southern Lake Michigan are similar in their community composition as well as their preference for substrates for cellular growth/metabolism. However, they differ in their gene expression profiles, with the offshore bacterioplankton showing higher gene expression for the uptake and degradation of compounds such as amino acids, sugars, peptides, nucleobases, urea, and inorganic phosphate. These differences in gene expression across Lake Michigan were observed in tandem with differing DOM quality between coastal and offshore regions, reflecting the importance of terrestrial subsidies in maintaining coastal productivity even after invasive dreissenid mussel proliferation in the lake. The results provide strong molecular-level evidence for substrate limitation in bacterioplankton of offshore Lake Michigan and also highlight the molecular composition of the preferred substrates in the community. Future research should focus on investigating how these trends in bacterial metabolism persist or change over time in this ecologically sensitive and valuable freshwater resource.

## Data Availability

All sequence data associated with this study have been deposited in the NCBI Sequence Read Archive under the accession number PRJNA1297788. The nucleotide sequences for the metagenome-assembled genomes used in this study are available from https://github.com/Aditchaudhary/Lake-Michigan-MAGs.

## SUPPLEMENTAL MATERIAL

Supplemental figures aem.00263-26-s0001.pdf Fig. S1 to S7.

Table S1 aem.00263-26-s0002.xlsx Summary metrics for metatranscriptomic libraries generated in this study.

Table S2 aem.00263-26-s0003.xlsx Relationship between specific transporter gene families and their broad substrate categories.

Table S3 aem.00263-26-s0004.xlsx Summary statistics for the metagenome-assembled genomes (MAGs) used for population tracking across Lake Michigan.

Table S4 aem.00263-26-s0005.xlsx MaAsLin2-based mixed effects model results evaluating variation in bacterial community gene expression in Lake Michigan as a function of the fixed effect 'Location' (nearshore v/s offshore, with nearshore as the reference).

Table S5 aem.00263-26-s0006.xlsx List of C/N/P cellular stress-marker genes used for data mining in this study, and their KEGG/COG database accession numbers.

ASM does not own the copyrights to Supplemental Material that may be linked to, or accessed through, an article. The authors have granted ASM a non-exclusive, world-wide license to publish the Supplemental Material files. Please contact the corresponding author directly for reuse.
