# Supplementary material for: Metatranscriptomics-based investigation of bacterial community dynamics across a dissolved organic matter gradient in southern Lake Michigan
Source: Appl Environ Microbiol. 2026 Mar 20;92(4):e00263-26. doi: 10.1128/aem.00263-26 (PMC13101498; doi:10.1128/aem.00263-26)
Supplement: Supplemental figures — Fig. S1 to S7. [file aem.00263-26-s0001.pdf]

Metatranscriptomics based investigation of bacterial community dynamics across a dissolved organic matter gradient in southern Lake Michigan

Adit Chaudhary[1*], Hui Lin[2], Laodong Guo[2], Rachel Poretsky[1]

[1]Department of Biological Sciences, University of Illinois at Chicago, Chicago, IL, United States of America

[2]School of Freshwater Sciences, University of Wisconsin-Milwaukee, Milwaukee, WI, United States of America

[*] Corresponding author. Email address: adit_chaudhary@rush.edu

Supplementary Figures

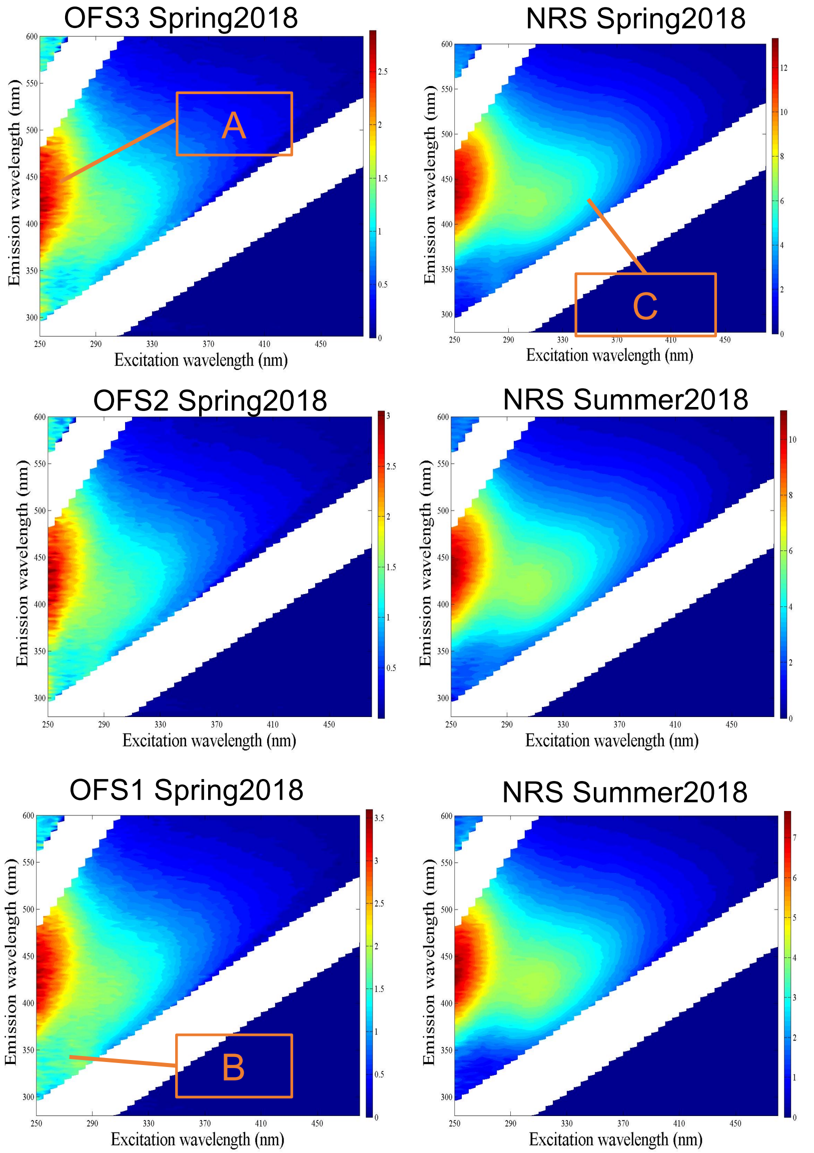

Figure S1: Characterization of fluorescent EEM spectra of DOM from nearshore and offshore Lake Michigan surface water, with major fluorescence peaks labeled, including Peak A corresponding to humic-like DOM, peak B to protein-like DOM, and peak C to terrestrially-derived humic-like DOM.

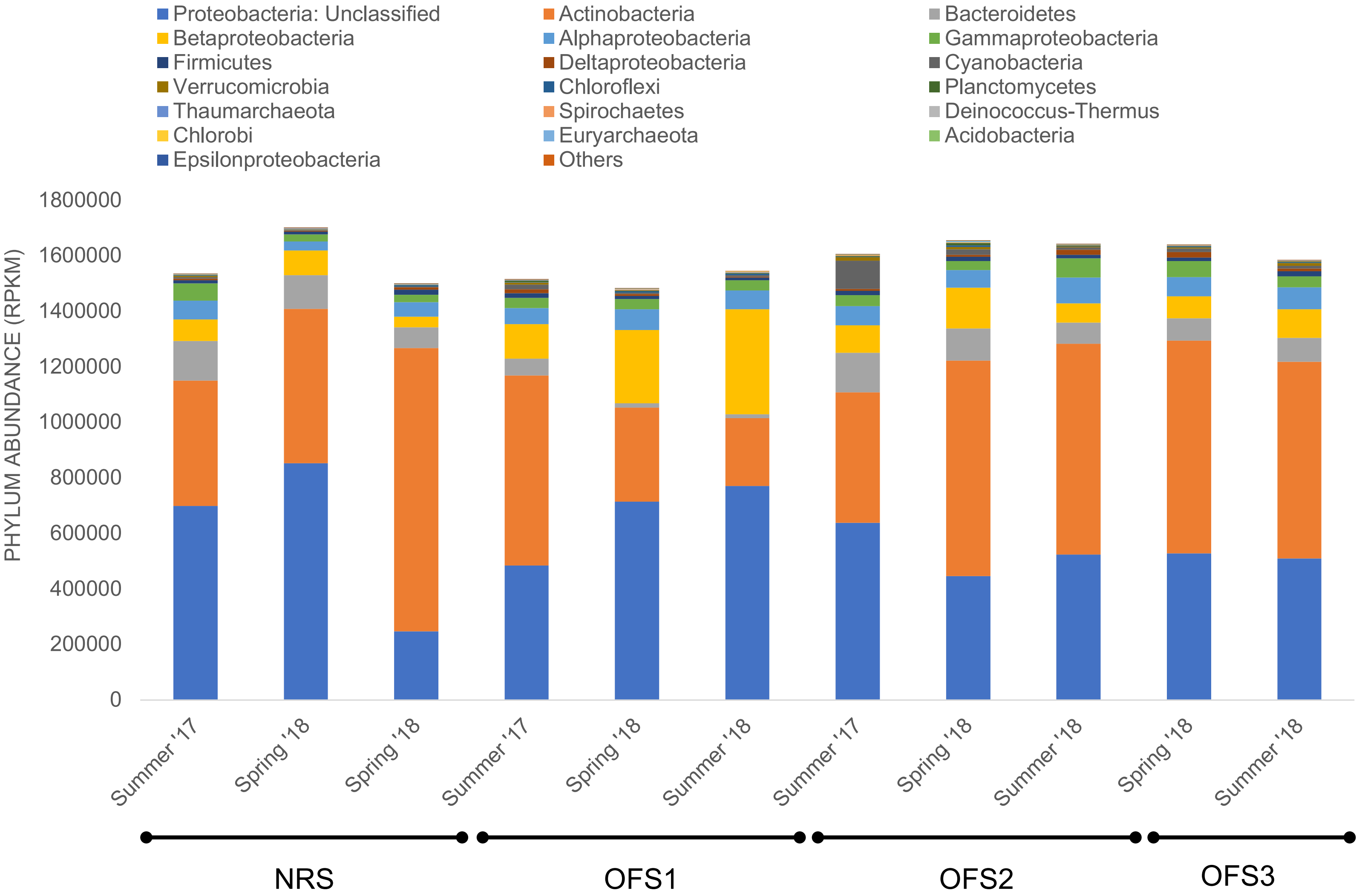

Figure S2: Barplot of metatranscriptome-based bacterial community composition at the phylum level (Proteobacteria subdivided into classes) across southern Lake Michigan. For each sample, transcript abundance was normalized using the RPKM formula (cDNA reads per kilo base gene per million mapped reads) prior to visualization.

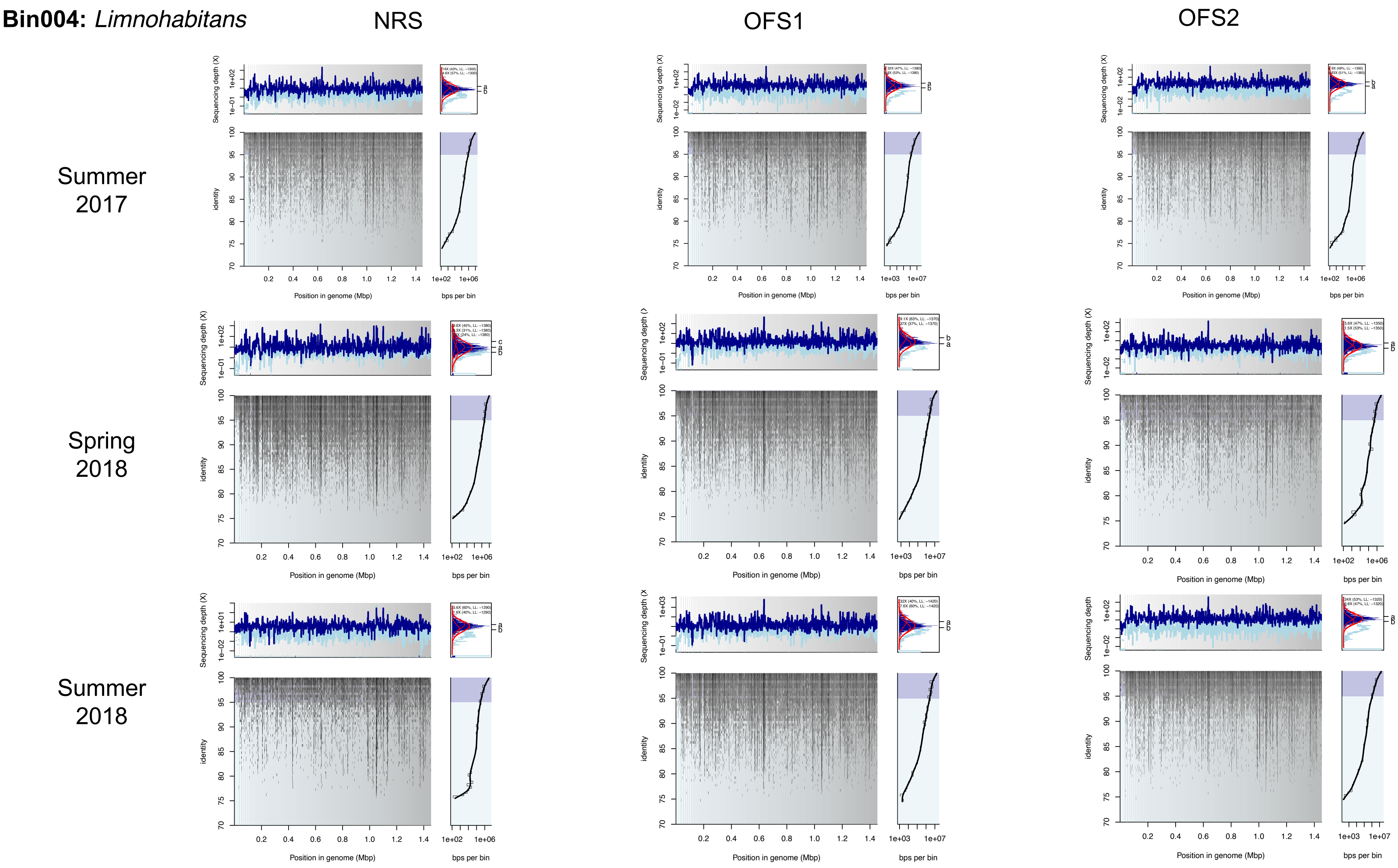

Figure S3: Read recruitment plots for MAG-based population LMS_bin004 (*Limnohabitans*) in nearshore (NRS) and offshore (OFS1 & OFS2) southern Lake Michigan. The coverage histogram (top left) in each plot shows coverage for the MAG in the corresponding Lake Michigan metatranscriptome from cDNA reads that match at > 95% nucleotide identity and > 70 bp in length (dark blue) as well as reads that match at > 70bp in length and < 95% nucleotide identity (light blue).

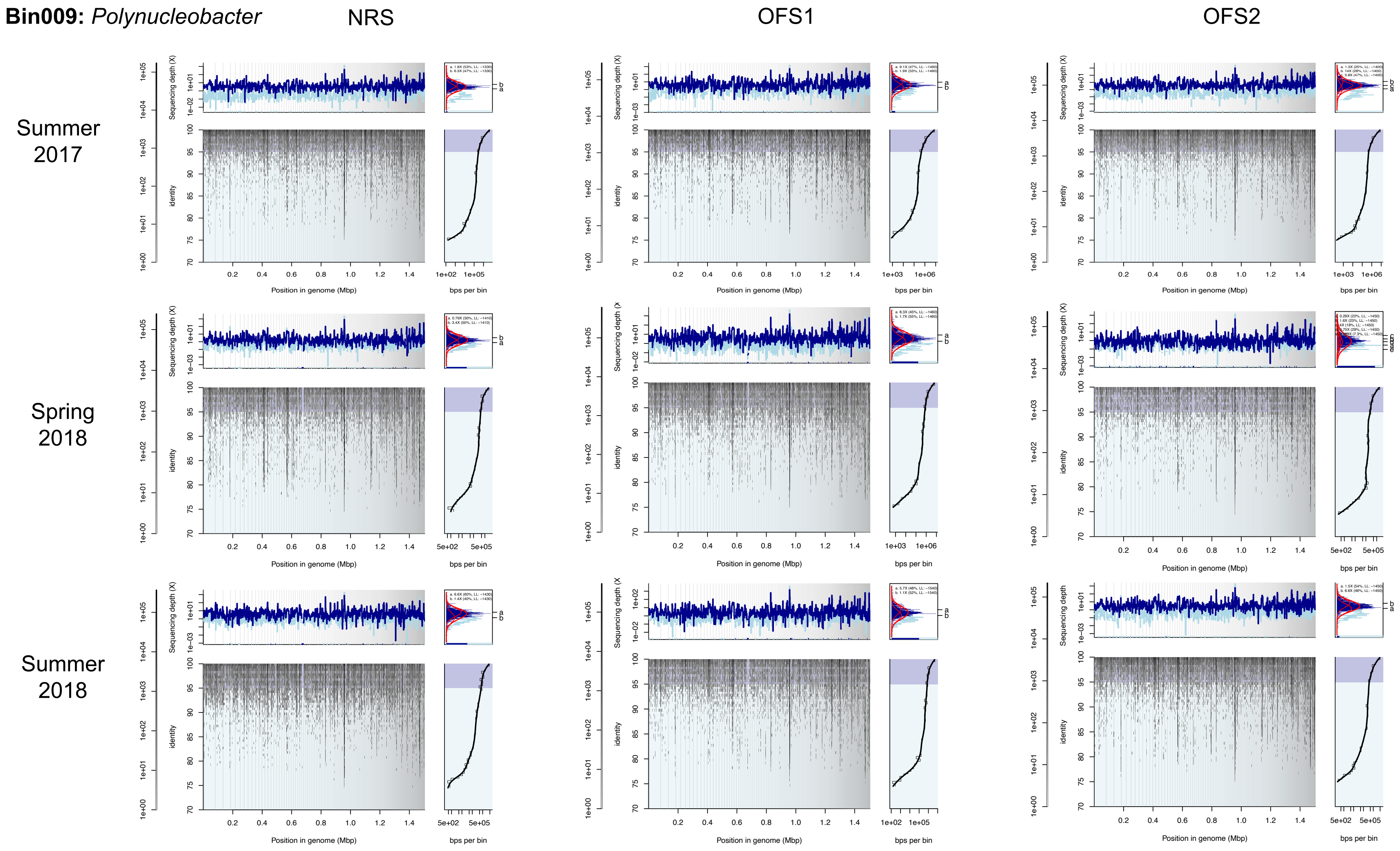

Figure S4: Read recruitment plots for MAG-based population LMS_bin009 (*Polynucleobacter*) in nearshore (NRS) and offshore (OFS1 & OFS2) southern Lake Michigan. The coverage histogram (top left) in each plot shows coverage for the MAG in the corresponding Lake Michigan metatranscriptome from cDNA reads that match at > 95% nucleotide identity and > 70 bp in length (dark blue) as well as reads that match at > 70bp in length and < 95% nucleotide identity (light blue).

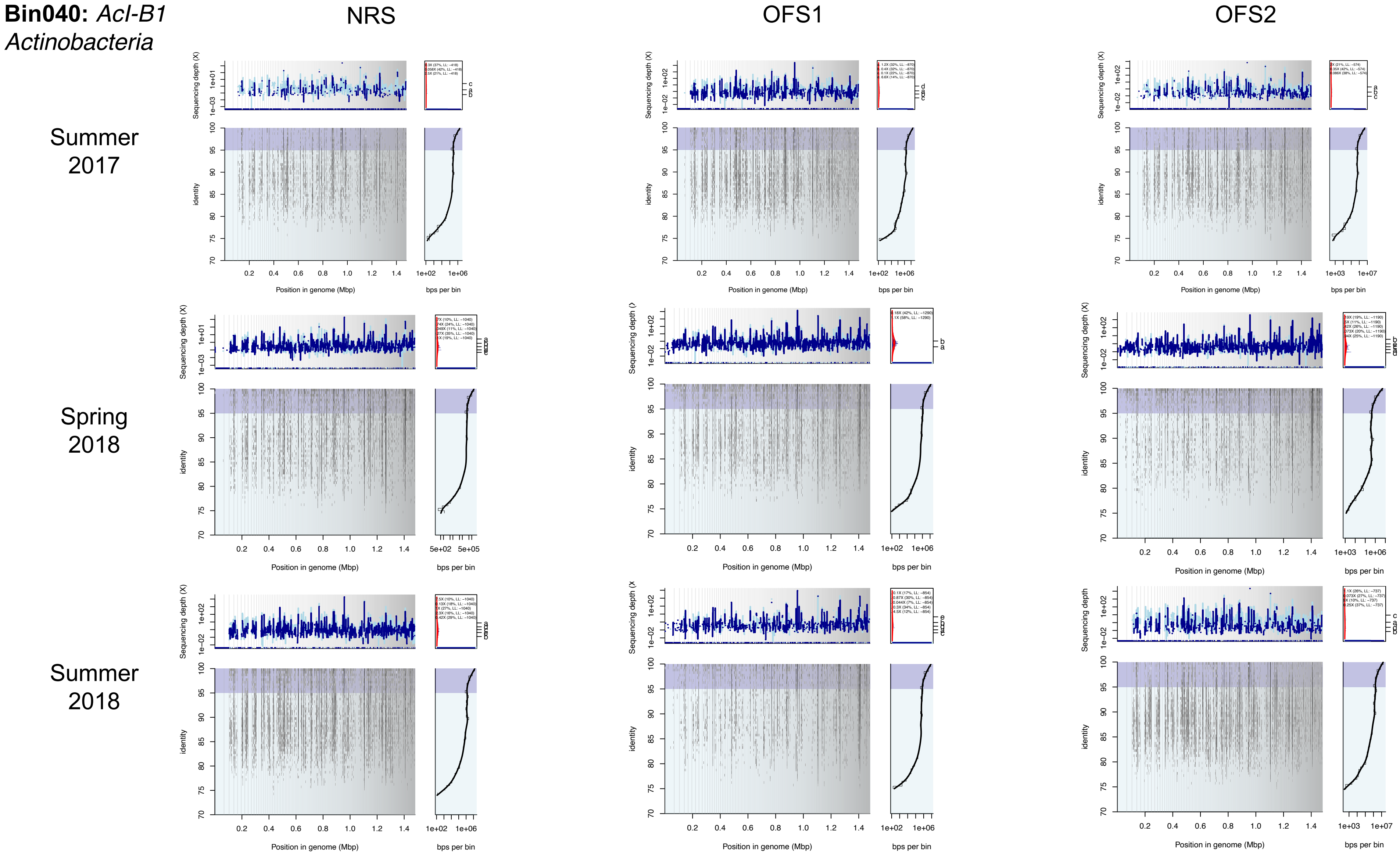

Figure S5: Read recruitment plots for MAG-based population LMS_bin040 (AcI-B1 Actinobacteria) in nearshore (NRS) and offshore (OFS1 & OFS2) southern Lake Michigan. The coverage histogram (top left) in each plot shows coverage for the MAG in the corresponding Lake Michigan metatranscriptome from cDNA reads that match at > 95% nucleotide identity and > 70 bp in length (dark blue) as well as reads that match at > 70bp in length and < 95% nucleotide identity (light blue).

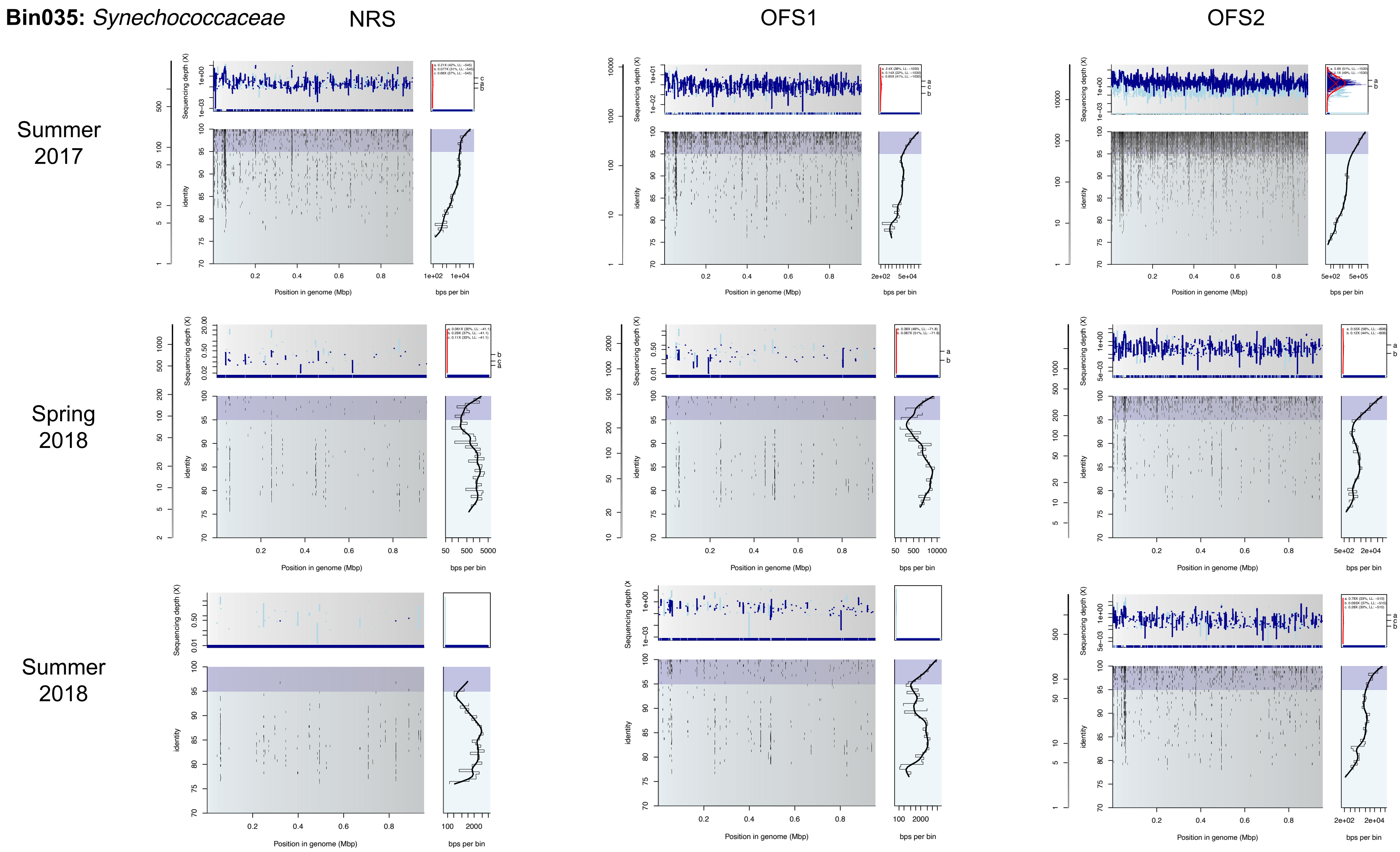

Figure S6: Read recruitment plots for MAG-based population LMS_bin035 (*Synechococcaceae*) in nearshore (NRS) and offshore (OFS1 & OFS2) southern Lake Michigan. The coverage histogram (top left) in each plot shows coverage for the MAG in the corresponding Lake Michigan metatranscriptome from cDNA reads that match at > 95% nucleotide identity and > 70 bp in length (dark blue) as well as reads that match at > 70bp in length and < 95% nucleotide identity (light blue). The recruitment plots (bottom left) show the individual reads mapping to the MAG at each position in the genome.

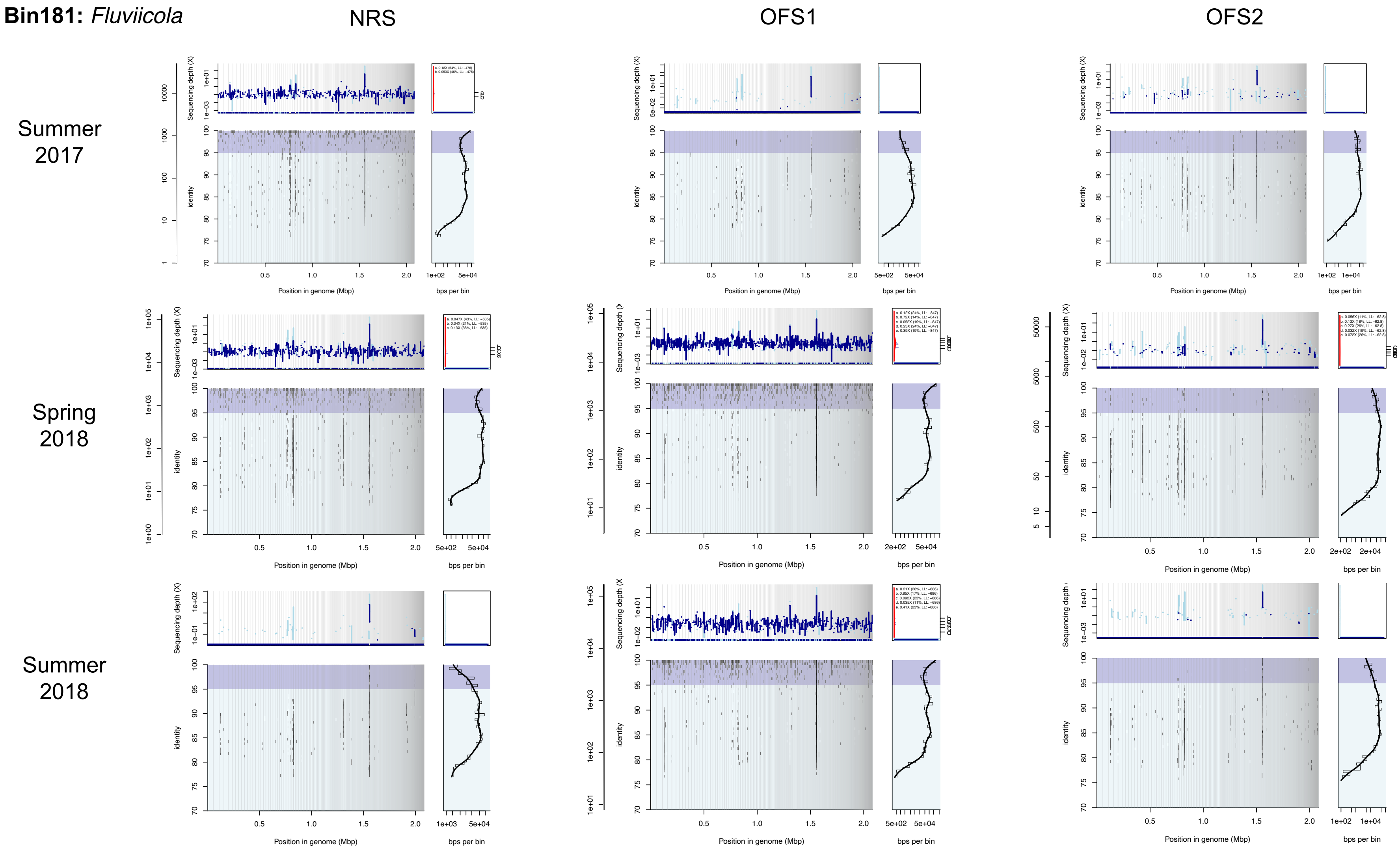

Figure S7: Read recruitment plots for MAG-based population LMS_bin181 (*Fluviicola*) in nearshore (NRS) and offshore (OFS1 & OFS2) southern Lake Michigan. The coverage histogram (top left) in each plot shows coverage for the MAG in the corresponding Lake Michigan metatranscriptome from cDNA reads that match at > 95% nucleotide identity and > 70 bp in length (dark blue) as well as reads that match at > 70bp in length and < 95% nucleotide identity (light blue). The recruitment plots (bottom left) show the individual reads mapping to the MAG at each position in the genome.
